# A model of see-saw nystagmus

**DOI:** 10.1007/s00415-026-13956-1

**Published:** 2026-06-26

**Authors:** Marcello Cherchi

**Affiliations:** Department of Neurology, The University of Chicago, 5841 South Maryland Avenue, Chicago, IL 60637 USA

**Keywords:** Nystagmus, See-saw nystagmus, Control systems analysis, Modeling, Superior colliculus, Interstitial nucleus of Cajal

## Abstract

See-saw nystagmus (SSN), and the possibly related hemi-see-saw nystagmus (hSSN), occur in certain forms of visual loss and in some brainstem lesions. These disparate lesions have made it challenging for investigators to arrive at a unified mechanism. Here we propose a model involving detection of peripheral retinal disparity in the superior colliculi (SC), which send inhibitory projections to the interstitial nucleus of Cajal (INC) that maintain calibration, and how loss of such calibration may provoke INC neurons (which are already mutually inhibitory with contralateral INC neurons) to develop self-inhibitory axo-dendritic autapses, resulting in a network configuration from which a pathologic Matsuoka oscillator can emerge and drive the alternating vertical and torsional movements characteristic of SSN and hSSN.

## Introduction

See-saw nystagmus (SSN), first described by Ernest Edmund Maddox [44] in a patient with bitemporal hemianopsia, is an ocular motor abnormality that occurs in some forms of visual loss and in some brainstem lesions. That an apparently singular ocular motor abnormality can result from such disparate lesions has understandably led some investigators to opine that, “The pathophysiological mechanism of see-saw nystagmus has remained obscure” [16], “It is concluded that the pathogenesis is unknown” [38], “the underlying mechanism of SSN is not precisely known” [69], “The exact mechanism of see-saw nystagmus remains uncertain” [81], etc.

Here we propose a model comprising a unified mechanism for SSN. The key features of this model include that:

1. The eye movement itself is not pathological, as it occurs in normal ocular counterroll (OCR), and is triggered by projections from the utricle and the vertical semicircular canals to the interstitial nuclei of Cajal. A neuron in one INC normally has mutually inhibitory projections (via the posterior commissure) with a neuron in the contralateral INC.
2. Switching from far-viewing OCR to near-viewing OCR results in peripheral retinal disparity because the visual axes are not parallel to the axis around which the head rotates in the coronal plane, so the far-viewing OCR response is modulated (reduced) during convergence in order to maintain stereopsis. It is plausible that this modulation is driven by inhibitory input from the superior colliculus (SC), which is the first structure in the visual pathways that has access to binocular information and therefore the first structure that can detect retinal disparity.
3. If the SC cannot detect retinal disparity (as occurs in peripheral visual loss from any cause), or if the SC projections to the INC are interrupted (such as from a midbrain lesion), then the INC is disinhibited and vulnerable to excitotoxicity.
4. INC neurons may, through neural plasticity, avoid excitotoxicity by acquiring additional inhibition, such as from a self-inhibitory axo-dendritic autapse.
5. When mutually inhibitory INC neurons additionally become self-inhibiting, then they can pathologically evolve into a central pattern generator—specifically, a type of half-center oscillator called a Matsuoka oscillator—which would result in the ocular motor patterns of SSN and hSSN.

### The eye movements at issue

The pattern of eye movements consists of:

1. Elevation and incyclotorsion of eye *A* synchronous with depression and excyclotorsion of the fellow eye *B*.
2. Return to baseline.
3. Depression and excyclotorsion of eye *A* synchronous with elevation and incyclotorsion of fellow eye *B*.
4. Return to baseline.
5. Repeat.

### The eye movements in physiologic circumstances: ocular counterroll

These vertical and torsional eye movements in question are not in themselves pathological; they occur as healthy physiologic responses in normal ocular counterroll (OCR), and comprise the expected vestibulo-ocular reflex response to head roll (rotation of the head in the coronal plane) during far-viewing.

The eye movements are a response to the vectorial component of gravity in the coronal plane; change in that vector (triggered by head roll) is detected by the utricle and the vertical (superior and posterior) semicircular canals, whereas static direction of that vector (when the head maintains a particular position in the roll plane) is detected by the utricle alone.

These physiologic vertical and torsional eye movements are orchestrated by signaling from the interstitial nuclei of Cajal (INC) [15]. The fact that these eye movements do not occur in microgravity [52] supports the claim that they are vestibularly driven, as opposed to a cervico-ocular response.

The continuous, oscillatory eye movements of SSN can be mimicked when a healthy individual slowly rocks the head laterally from side to side, effectively swinging the vector of gravity back and forth between (1) inferiorly and rightward, and (2) inferiorly and leftward.

Note that during far-viewing, when there is maximal divergence, the optical axes are parallel to the naso-occipital axis, and this parallelism is maintained during OCR with far-viewing. In contrast, during OCR with near-viewing, when there is some degree of convergence, if one were simply to pull the optical axes to a point half-way in between them, “the optical axes are not parallel to the axes around which the head and eyes are rotating” [54], and consequently there would be greater cyclodisparity, and the corresponding retinal disparity would be most pronounced in the peripheral (especially the temporal) visual fields [29]. This greater peripheral retinal cyclosdisparity can be quantified [34].

### The eye movements in pathologic circumstances: see-saw nystagmus

What is pathological in SSN is that these eye movements occur spontaneously and in an oscillating pattern, rather than as a vestibularly-driven response to a change in the gravitational vector.

Good diagrams of the eye movements in SSN can be found in Daroff [16] and Druckman and colleagues [21]. Good videos of SSN can be found in Rudich and Lesser [67], and Jeong and colleagues [39].

In see-saw nystagmus (SSN), the entire sequence of eye movements (#1 – #4 above) is pendular (the velocity is symmetrically sinusoidal). In hemi-see-saw nystagmus (hSSN), the eye movement velocities are not sinusoidally symmetrical; rather, in one direction (say steps #4 and #1) they are slow, whereas in the opposite direction (say steps #2 and #3) they are rapid.

### Circumstances in which see-saw nystagmus occurs

SSN tends to occur in specific circumstances, including:

1. Visual abnormalities, such as:
  Bitemporal hemianopsia, such as from acquired optic chiasm lesions, most commonly due to compression from a tumor [20, 59]. It is noteworthy that if the vision loss is reversed (such as by extirpation of a tumor that had been compressing the optic chiasm), then SSN may cease [40, 43, 48, 59, 73].
  Individuals with congenital achiasma [18, 42, 57, 61] or in whom the chiasm is present yet retinal fibers do not decussate [1, 2, 6], or with septo-optic dysplasia [17, 67]. Note these individuals are abnormal in that the (retrochiasmic) optic tracts carry signals only from the ipsilateral retina (since the fibers from the nasal retina of the fellow eye do not decussate), though they do not have any visual field defects.
  Peripheral visual loss as part of broader pan-retinal blindness, such as retinitis pigmentosa [9, 12], rod-cone dystrophy [52] and infectious choroiditis [66].
2. Various brainstem lesions, predominantly involving the midbrain, such as:
  See-saw nystagmus
      “Brainstem vascular disease involving the midbrain and probably the immediate rostral structures” (Daroff 1965).
      “Giant cavernoma localized at the left meso-diencephalic region” (Bassani and Marzoli 2013).
      “Midline meso-diencephalic junction” (Endres et al. 1996).
      “Unilateral mesodiencephalic lesion” (Halmagyi and Hoyt 1991).
  Hemi-see-saw nystagmus
    “Unilateral meso-diencephalic lesion” (Halmagyi et al. 1994).

### Circumstances in which see-saw nystagmus does not occur

SSN does *not* occur in the following circumstances:

- Blindness from central retinal deficits, such as in macular degeneration.
- Cortical blindness, such as from occipital infarcts.
- Reduction or absence of vestibular input, as occurs in vestibular loss or in microgravity.

### Candidate anatomical substrate (normal neuroanatomy)

Normal, vestibularly-driven ocular counterroll requires integrity of the following neuroanatomical pathways:

- The utricle detects the direction of whatever component of the vector of linear acceleration lies in the coronal plane (such as from gravity).
- The vertical (superior and posterior) semicircular canals detect rotational acceleration in the coronal plane (such as from lateral heat tilt); they do not detect static linear acceleration.
- Those inputs encoding vectorial components of acceleration in the coronal plane arrive at the interstitial nucleus of Cajal (INC). Inputs from the utricle arrive directly (monosynaptically) at the magnocellular medial vestibular nucleus (MVmc) [58] (page 717, Fig. 17.2). Inputs from the vertical semicircular canals arrive at other vestibular nuclei (superior, lateral, inferior and descending), but eventually reach the MVmc through disynaptic projections.
- Some neurons in the magnocellular medial vestibular nucleus send excitatory projections to the contralateral interstitial nucleus of Cajal (INC); see [25, 26, 41] and [58] (page 724, Fig. 17.6).
- The INC sends projections encoding eye position to the trochlear and ocular motor nuclei to coordinate contraction and relaxation of the relevant superior and inferior recti and obliques [58] (page 727, Fig. 17.8), resulting in vertical and torsional eye movements.

### Mechanisms of oscillation

Neural mechanisms for generating oscillations were first proposed by Thomas Graham Brown [11], and are now broadly referred to as central pattern generators (CPG) [35]. Selverston and colleagues [71] explain that:

“There are two ways that neuronal oscillators could work: one or more cells… would have the property of endogenous bursting (cell-driven oscillators), or else the network itself would produce bursts as a result of synaptic interactions (network oscillators)” [71].

Regarding cell-driven oscillators:

“If a cell can be completely isolated from any synaptic input… and the cell is still able to produce bursting pacemaker potentials, it can be considered a true endogenous burster” [71].

Regarding network oscillators:

“In circuits without endogenous bursters, oscillatory activity can be generated as a function of the connections alone” [71].

The pattern of eye movements in SSN would more plausibly be driven by gradual alternation of activity between the two sides (and specifically, by a network oscillator whose paired components are anti-phase locked), rather than by activity of only one side that alternates between on and off (i.e., an isolated burst oscillator). As such the resulting behavior in SSN would be an emergent property (albeit a maladaptive one) of the network—in other words, a system-level pattern that could not be predicted from the properties or behaviors of any single element in isolation [64].

This kind of anti-phase locked alternating activity can arise from a CPG called a half-center oscillator ([8, 47]). A parsimonious and biologically plausible oscillator was proposed by Kiyotoshi Matsuoka [50, 51], and has been used to model various normal biomechanical processes such as ambulation [28], lateral undulation [62, 68] and mastication [79]. A Matsuoka oscillator is very simple in that it only requires: (1) that there be two neurons; (2) that those two neurons are mutually inhibitory; and (3) that each of those two neurons is also self-inhibitory. We will discuss implementation of a Matsuoka oscillator below.

### A model of see-saw nystagmus

We have now outlined the necessary neuroanatomical components and neurophysiologic concepts to assemble a model.

### Normal physiology needed for SSN

In normal individuals the superior colliculus is the first neuroanatomical structure in the visual pathway that has access to binocular information [74]. Animal studies suggest that it is the earliest point in the visual pathways at which binocular disparity can be detected [3–5, 10, 19, 30, 55, 77], and is thus the first structure that could trigger responses to maintain stereopsis [77]. Note that the granularity of disparity detection in the superior colliculus is sometimes described as “coarse” [55]; this contrasts with the more refined processing of disparity that occurs further downstream in visual processing, such as at higher levels of the visual cortex for calculating stereopsis [53].

Some neurons in the intermediate layer of the superior colliculus send inhibitory projections to the ipsilateral interstitial nucleus of Cajal; see [33] and [58] (page 774, Fig. 19.10). If some of these signals reflect detection of retinal disparity, then this signal probably comprises a tonic level of inhibition, whose frequency is directly proportional to the degree of peripheral retinal disparity.

The INC receives inputs from multiple structures [25], but the ones relevant to this discussion are that it receives *excitatory* (vestibular) signals from the contralateral magnocellular medial vestibular nucleus, and *inhibitory* (peripheral retinal disparity-sensing) signals from the intermediate layer of the ipsilateral superior colliculus.

The INCs on each side of the brainstem communicate via the posterior commissure. Some of these connections comprise mutually inhibitory projections [76].

### Pathophysiology needed for SSN

If inhibitory projections from the SC to the INC are impaired (either because the SC fails to detect peripheral retinal cyclodisparity, or because the projection from the SC to the INC is interrupted by a lesion), then in this relatively disinhibited state, the INC is at risk for developing excitotoxicity. In order to offset and avoid such excitotoxicity, INC neurons could get additional inhibition from two sources: (1) sprout an axo-dendritic self-inhibitory autapse [60]; or (2) recruit an inhibitory interneuron. Although both are possible, the former (an autapse) is simpler (in that it entails a monosynaptic rather than disynaptic circuit) and thus more plausible.

Earlier we discussed that Matsuoka oscillators have been used to model normal physiologic processes. Matsuoka oscillators have also been used to explain pathological processes, including certain saccadic oscillations [72], and we will invoke that mechanism here.

To review the features required for a Matsuoka oscillator that we mentioned earlier and how they are met in the current model:

1. There are two neurons. In this case a pair, with one in each INC.
2. The two neurons are mutually inhibitory. In this case, the paired INC neurons are normally mutually inhibitory via connections through the posterior commissure.
3. Each neuron is also self-inhibitory. In this case this occurs when each INC neuron develops self-inhibition in order to avoid excitotoxicity (resulting from the reduction or absence of inhibition from the SC).

Paired neurons with features 1 and 2 that under normal circumstances were not intended to form a CPG can pathologically *become* a Matsuoka oscillator when feature 3 arises, after which they will eventually reach a Hopf bifurcation [36, 37], and destabilize into a limit cycle that is rhythmogenic—in this case, producing an oscillatory output. Other models of ocular oscillations have postulated that such emergence of rhythmogenicity can arise after removal of an external input (excitatory or inhibitory) destabilizes a circuit [56, 72].

Note that *this oscillatory activity arises from the INC themselves*. It occurs independently of any saccadic velocity commands from the rostral interstitial nucleus of the medial longitudinal fasciculus (riMLF), and independently of smooth pursuit commands from cerebellar flocculus and paraflocculus; it occurs even when input from the vestibular nuclei is static or absent.

### Simulations using this model

**Fig. 1:**
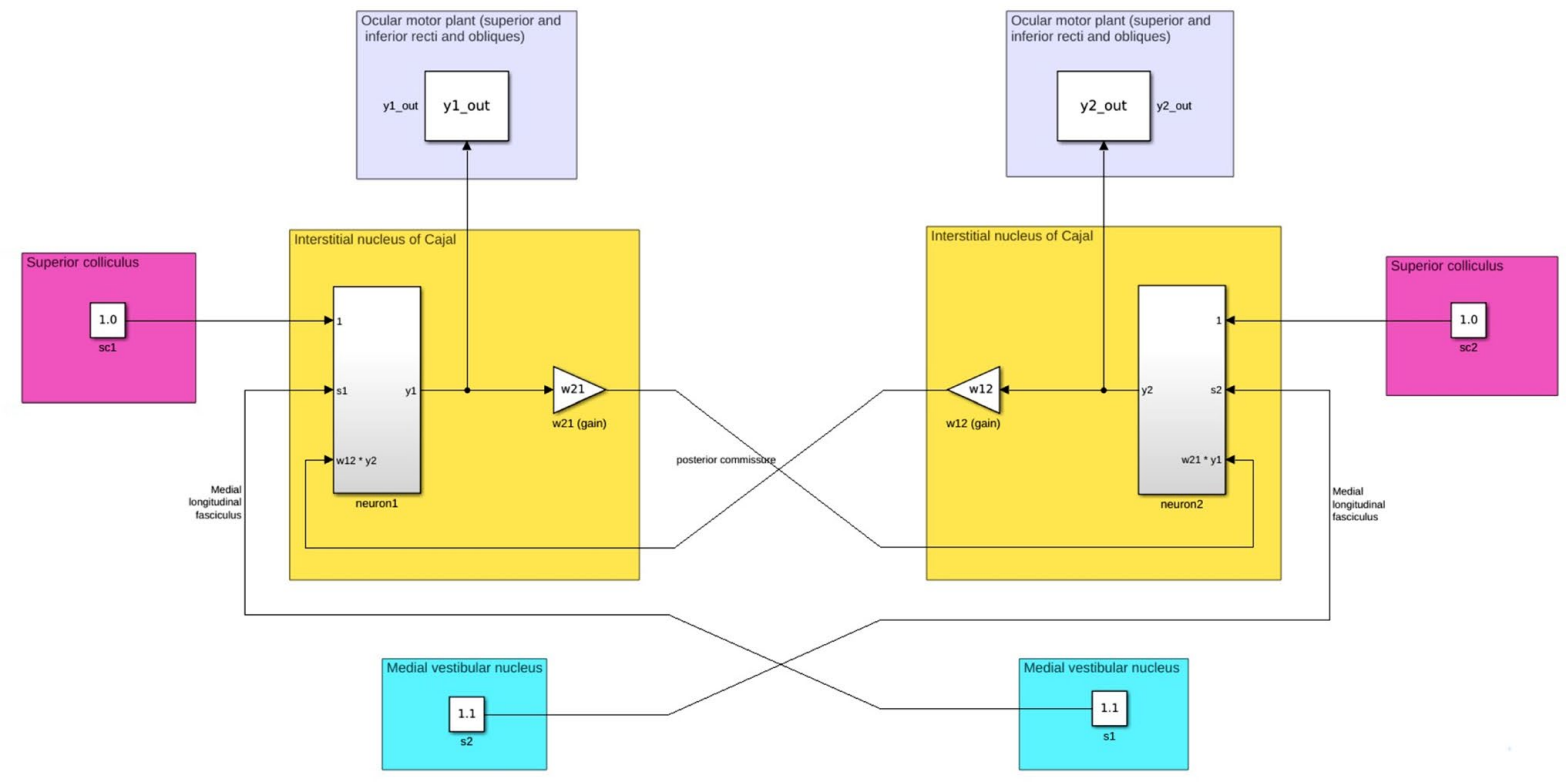
Model of see-saw nystagmus, developed in Matlab’s Simulink^®^. The core of this system consists of the interstitial nuclei of Cajal (INC). Each INC neuron receives inhibitory input from the ipsilateral superior colliculus and excitatory input from the contralateral magnocellular component of the medial vestibular nucleus (MVmc). Each INC also projects to the ocular motor plant. In the healthy state, each INC neuron is mutually inhibitory with the contralateral INC neuron. In the pathologic state, each INC neuron is also self-inhibitory. In that pathologic state, this system can function as a Matsuoka oscillator, maladaptively becoming rhythmogenic

We developed this model starting with the Matsuoka oscillator presented in Xu and colleagues [79], which was coded into Matlab’s Simulink® by Manurung [46]. We modified this to reflect that each neuron receives excitatory input from the contralateral medial vestibular nucleus, receives inhibitory output from the ipsilateral superior colliculus, and sends output to the various ocular motor nuclei (specifically the oculomotor nuclei and trochlear nuclei, conflated here for simplicity) to control the ocular motor plant (Fig. 1). Since the INC output encodes vertical and torsional eye position, the oscillatory output generated by this model would drive eye movements of SSN (Fig. 3).

The overall system, with two mutually inhibitory neurons, is shown in Fig. 1.

**Fig. 2:**
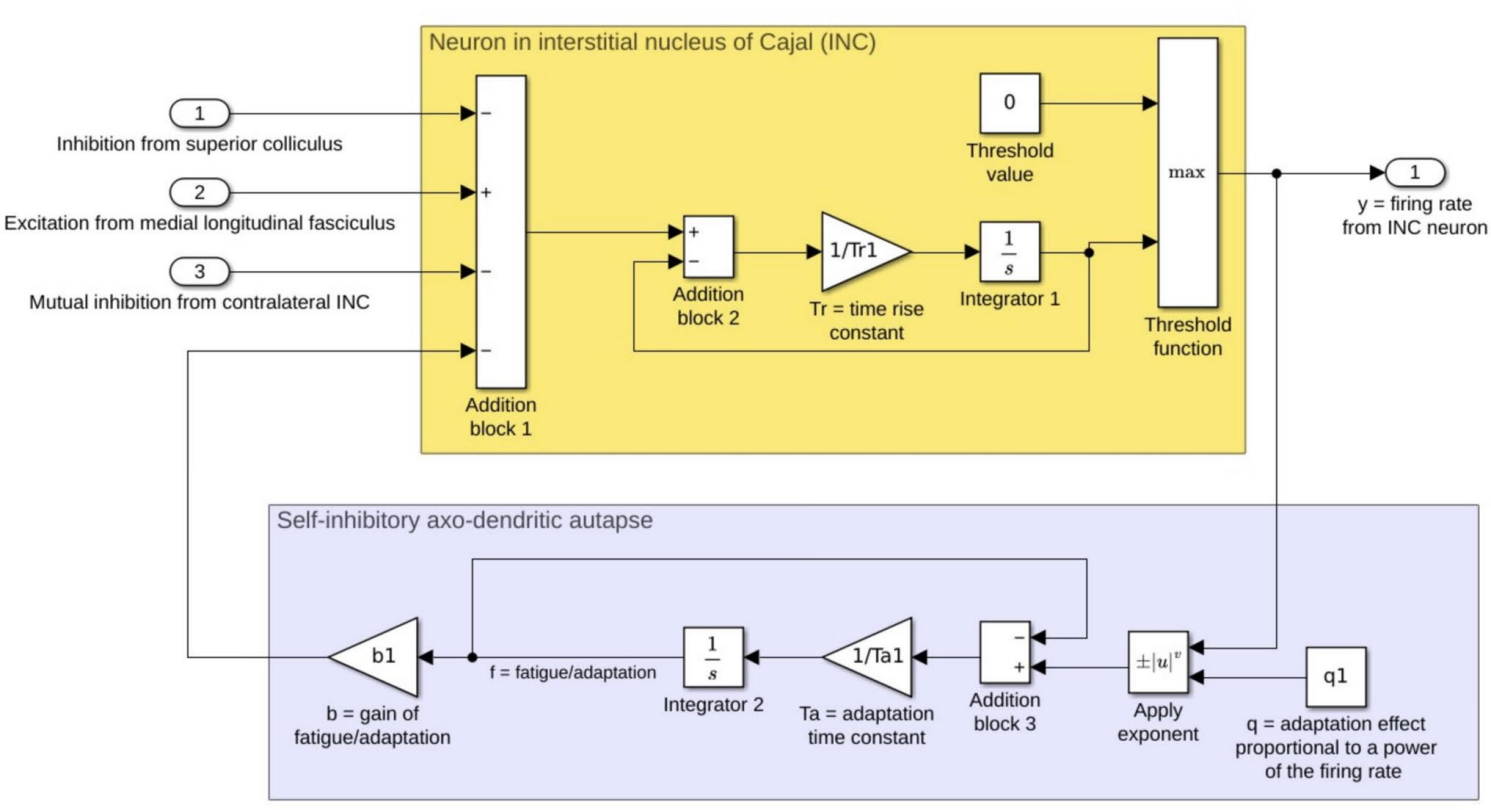
Model of one INC neuron. The components in the yellow box are the normal neuron. The components in the purple box comprise a pathologic auto-inhibitory axo-dendritic autapse

An example of each individual neuron is shown in Fig. 2.

**Fig. 3:**
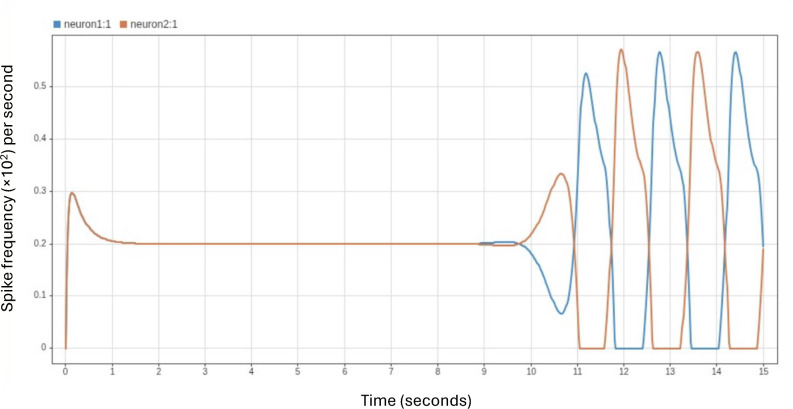
Pathologic oscillatory output resembling see-saw nystagmus (SSN), generated in Matlab’s Simulink®. The X axis represents time. The Y axis represents frequency of spikes of an INC neuron on a given side. Note that the firing rate of INC neuron 1 and INC neuron 2 are anti-phase locked, and symmetrical in the sense that that the peak frequency of firing of one neuron alternates in a regular pattern with that of the other neuron

Figure 3 shows an oscillatory output generated by this model resembling SSN.

**Fig. 4:**
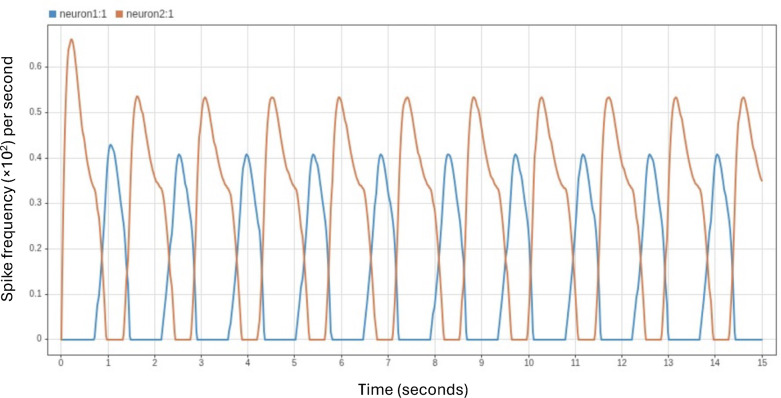
Pathologic oscillatory output resembling hemi-see-saw nystagmus (hSSN), generated in Matlab’s Simulink®. The X axis represents time. The Y axis represents frequency of spikes of an INC neuron on a given side. Note that the firing rate of INC neuron 1 and INC neuron 2 are anti-phase locked, but asymmetrical in that the frequency of neuron 2 hits a higher peak and for a longer duration than that of neuron 1. This pattern emerges when the adaptation constant of INC neuron 1’s self-inhibitory axo-dendritic autapse is diminished

Figure 4 shows an oscillatory output generated by this model resembling hSSN. On one side (neuron 1) the adaptation constant of the self-inhibitory axo-dendritic autapse has been shortened, resulting in a pattern of activity that is anti-phase locked, but asymmetrical in peak frequency and duration.

### Notable features of this model

This model provides a plausible mechanism to explain the following features of SSN:

- SSN *should* occur when there are deficits from peripheral retinal input (either due to retinal damage, or due to afferent pathway interruption at the optic chiasm), or midbrain lesions that interrupt projections from the SC to the INC.
- Cortical blindness should *not* cause SSN because retinal disparity has already been detected by the SC (earlier in the visual pathway).
- Monocular blindness should *not* cause SSN because there is still retinal disparity (blind eye ≠ sighted eye).
- Vestibular weakness should *not* cause SSN because SC inhibitory projections to the INC are preserved (and are not opposed by any excitatory vestibular input).
- Modifications in individual model parameters could correspond to the density of expressed ion channels in neuronal cell membranes. Modest modifications can manifest as:

1. Variations in the frequency of oscillation in SSN, which reflects findings in actual patients.
2. Asymmetry in the velocity of sinusoidal oscillations — in other words, generation of hemi-see-saw nystagmus. For example, the tracing in Figure 4 shows the result of diminishing the adaptation constant of the INC neuron on one side.

### Questions about this model

There are several questions about this model that merit scrutiny. We discuss three of these.

### Why does SSN develop slowly?

Many lesions resulting in SSN evolve slowly (e.g., gradual compression of the optic chiasm by a tumor; slow progression of retinitis pigmentosa or rod-cone dystrophy), which perhaps partly accounts for the slow emergence of SSN. However, SSN also develops slowly even after abrupt lesions, such as after traumatic brain injury [22, 80], or after pituitary apoplexy with abrupt compression of the optic chiasm. Probably the most important factor explaining the pace at which SSN emerges is that the relevant neuroplastic process (sprouting of self-inhibitory axo-dendritic autapses) is slow. Thus, even with an acute lesion (such as a midbrain infarct, which should abruptly reduce SC inhibition of the INC), it takes time for the maladaptive self-inhibition to build up in the INC to the point that would enable pathologic rhythmogenesis.

### What destabilizes the circuit to initiate oscillation?

Once the criteria for a Matsuoka oscillator are met, as long as the parameters on each side are exactly equal, the oscillation cannot begin, so what destabilizes (“kicks off”) an asymmetry that initiates the oscillation? The factors that initiate oscillation in the computational model are probably different than the factors that initiate oscillation in a true biological system, and this distinction merits discussion because it is one way in which the computational model may not satisfactorily explain the true biological system.

The computational model is constrained by serial architecture, so when sequential processing slightly changes one side (say SC signaling on one side), then that change will provoke immediate downstream consequences before the serial processing ever even reaches the contralateral SC. In other words, the asymmetry that arises in the computational model is an artifact resulting from the architecture of necessarily sequential processing.

In contrast, the biology of real neurons comprises truly parallel processing—but it faces different problems, namely that (1) there is probably never exact symmetry to begin with, and (2) there is stochastic variability in neural activity. The reduction of SC inhibitory signaling is slow because it is the downstream consequence of gradual degradation of peripheral vision (e.g., over years in rod-cone dystrophy; over months with optic chiasmal lesions), and this degradation is neither perfectly smooth nor perfectly symmetrical. Perhaps early in the disease process (when the signaling is just starting to degrade), the absolute difference (between the two sides) is proportionally small; however, as the degradation progresses there will be a “floor effect,” below which any differences between the two sides, even if small in absolute terms, are proportionally larger, and as that asymmetry advances and becomes more consistent, the likelihood of surpassing a Hopf bifurcation (unleashing pathological rhythmogenesis) increases.

### Can this model generate less regular waveforms?

**Fig. 5:**
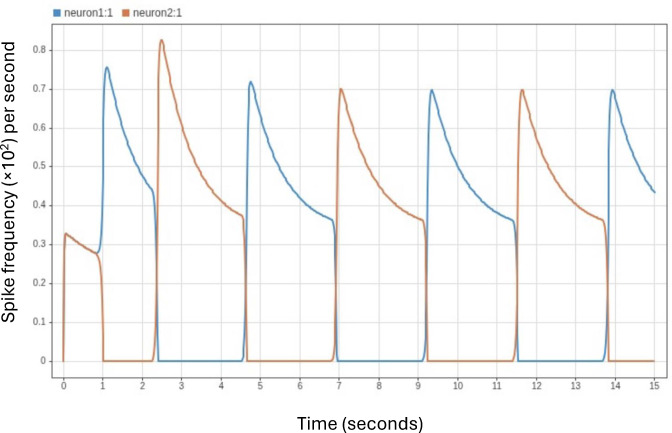
Pathologic oscillatory output from this model, generated in Matlab’s Simulink®, showing anti-phase-locked behavior that is not in a smoothly sinusoidal, pendular pattern. This pattern emerges when the gain of the self-inhibitory autapse is diminished. The X axis represents time. The Y axis represents frequency of spikes of an INC neuron on a given side

Reviewers pointed out that unlike idealized textbook depictions, the vertical and torsional components of the eye movements in real cases of SSN are not always smoothly sinusoidal and pendular. This model can capture such behavior. To provide just one example, Fig. 5 shows the waveforms that result from decreasing the gain of the self-inhibitory autapse on both sides.

### Predictions from this model

As long as there is still some inhibitory input from the SC, this model predicts that vestibular suppression may help SSN, because bringing the magnitude of the excitatory input to the INC (consisting of vestibular signals from the magnocellular component of the medial vestibular nuclei) below the magnitude of the inhibitory input to the INC (consisting of retinal disparity signals from the SC) should enable the inhibitory input (however small) to keep the INCs “calibrated.” This is a very counterintuitive prediction, in the sense that clinicians do not usually expect a “centrally” mediated ocular motor abnormality to be affected by a primarily “peripherally” acting drug. Many patients with SSN attempt (or their clinicians prescribe) a trial of a vestibular suppressant (such as meclizine or dimenhydrinate), but they typically terminate this after a few weeks due to lack of improvement. It may be that a longer trial of vestibular suppression is needed before the maladaptive self-inhibitory axo-dendritic autapses “un-wire” themselves. Although interesting, this prediction might not have practical clinical implications, since the symptoms from chronic pharmacologic vestibular suppression may not be a favorable exchange for the reduction in SSN; nevertheless, since patients with SSN typically describe the visual experience as the most disturbing symptom, it is possible that this “trade off” (less visual symptoms, yet some unsteadiness from vestibular suppression) would be experienced as preferable.

### Limitations of this model

A relatively smaller number of cases describe SSN and hSSN arising from other lesions, such as in the thalamus [78], pons [14], “lower brain-stem” [49], and cervico-medullary junction [81]. Such apparently exceptional cases should be interpreted with caution for several reasons. First, SSN sometimes occurs in conjunction with other ocular motor abnormalities [14, 23, 39, 45, 65, 70], or in a sequence with other ocular motor abnormalities [13]. Second, some reported cases have insufficient anatomical information, such as no imaging [24, 38], low-quality imaging [78], or no autopsy. It may still be able to incorporate some of these apparent exceptions into our model; for example:

- See-saw nystagmus(SSN):
  - Thalamic lesion:
    - Williams and colleagues described SSN in a patient with “Area of low density in the left thalamus consistent with an infarct” [78]. However, the imaging quality (early CT) in this study was poor, and although it does show an area of low density in the thalamus, it probably could not confidently exclude a small midbrain lesion. It may nevertheless be feasible to incorporate this into the model. For example, there is some animal evidence of thalamofugal projections to the superior colliculi [63], and it is conceivable that disrupting such thalamo-collicular projections (by lesioning the thalamus) could change the collicular processing of retinal disparity.
  - “Lower brain-stem” lesion:
    - Mastaglia and colleagues described SSN in a patient with a “Lower brain-stem infarction” [49]. This report was before advanced imaging was available, so the anatomical information is probably incomplete. Some features reported in this case, such as a left Horner syndrome, raise the possibility that a midbrain lesion was also present.
    - Zimmerman and colleagues reported SSN in a patient with a Chiari malformation with herniation of the cerebellar tonsils through the foramen magnum [81]. It may be feasible to incorporate this into the present model. For example, since there are projections from the cerebellar dentate and fastigial nuclei to the interstitial nucleus of Cajal [27], it is possible that cerebellar damage (from a Chiari malformation) interferes with these pathways, and thus with cerebellar modulation of activity in the INC. (See below regarding other candidate models.)
- Hemi-see-saw nystagmus (hSSN):
  - Pontine lesion. Choi and colleagues reported hSSN in a patient with a dorsomedial pontine infarction [14]. However, this case involved additional ocular motor abnormalities (such as internuclear ophthalmoplegia).

### Other candidate models

Other mechanisms of SSN are reasonable to consider. We shall review several here, noting specific features that make them seem less plausible.

### Oscillator is in the INC, but disinhibition is due to a cerebellar lesion

Cerebellar efferent projections are inhibitory, and some of these pathways influence most of the structures we have discussed (vestibular nuclei, INC, SC). Since SSN has been reported in what appear to be purely cerebellar lesions, it is reasonable to consider a model focusing on the cerebellum—for example, could reduction/ablation of cerebellar input to the INC result in disinhibition, leading to the pathological emergence of the same Matsuoka oscillator described above? In principle, this is certainly possible. However, in order to explain how peripheral visual loss causes SSN, such a model would need to posit a more complex pathway (SC to cerebellum to INC) with a larger number of synapses. Thus, although possible, such a model’s greater complexity makes it less plausible.

### Oscillator is in the SC

Another potential anatomical source of oscillation would be the SCs themselves. Studies show that the superior colliculi are connected by inhibitory and excitatory projections through the intercollicular commissure (commissure of the superior colliculi) [75]. The main problem for this model is that in order for mutually inhibitory SC neurons to become oscillators, each would additionally need to develop self-inhibition, whose trigger would be pathological removal of an incoming inhibitory signal, but the origin of such an inhibitory signal is unclear (retinal input, for example, is excitatory).

### Oscillator is in the vestibular nuclei

Yet another potential anatomical source for oscillation would be the vestibular nuclei. Since the vestibular nuclei on both sides communicate through robust commissural connections, could the paired cells in the magnocellular medial vestibular nuclei (some of which are mutually inhibitory) develop self-inhibition, and thus lead to the pathological emergence of a Matsuoka oscillator? Although possible, such a model faces at least two problems. First, if such a model were to take into account peripheral retinal disparity, then the simplest pathway would be a projection from the SC to the medial vestibular nuclei, but there is no evidence for such a pathway (at least not for a monosynaptic one). Second, such a model relies on the abnormal oscillatory signal being generated in the vestibular nuclei themselves, and those signals will eventually reach the thalamus and vestibular cortex, thereby leading to the patient’s false perception of rocking—but clinically these patients usually do not experience such vestibular illusions; rather, they describe the visual symptoms (oscillation) as the most intrusive.

## Summary and discussion

See-saw nystagmus (SSN) and hemi-see-saw nystagmus (hSSN) occur in certain forms of visual loss (impaired peripheral vision, particularly bitemporal visual loss) and brainstem lesions (particularly midbrain). The eye movement itself is not pathological, as it can occur in normal ocular counterroll (OCR), but its spontaneous occurrence *is* pathological. We proposed a model that unifies the appearance of SSN/hSSN in these disparate etiologies. The mechanism is:

1. The superior colliculus (SC) is the earliest point in the visual pathway that can detect retinal disparity.
2. Inhibitory projections from the SC to the interstitial nucleus of Cajal (INC) maintain calibration of INC.
3. Some of those INC neurons are normally mutually inhibitory with contralateral INC neurons via the posterior commissure.
4. Impairing peripheral visual input (such as from a chiasmal lesion) or impairing the SC-to-INC projections (such as from a midbrain lesion) decreases inhibition of the INC, some of whose cells, in order to avoid excitotoxicity, develop self-inhibitory axo-dendritic autapses.
5. Those INC neurons which are now self-inhibitory and mutually inhibitory meet criteria for a Matsuoka oscillator and can become rhythmogenic, developing anti-phase-locked oscillation that drives the alternating vertical and torsional eye movements of SSN and hSSN.

Modest variations in model parameters (perhaps corresponding to differentially expressed concentrations of ion channels in individual neurons) can produce SSN or hSSN, and can produce different frequencies of oscillations, as occur in real clinical cases.

We then discussed why SSN/hSSN develops slowly (whether the inciting lesion is acute or gradual), how the oscillation is initiated, and how this model can capture more irregular patterns (i.e., eye movements that are not smoothly sinusoidal and pendular). We reviewed some apparently exceptional cases and how it may still be possible to incorporate these into our model. We concluded with a discussion of several other possible models and why they seem less plausible than the one we have proposed.

## Data Availability

All relevant data are contained within the article. Further inquiries can be directed to the corresponding author.
